# Exploring Burnout Dimensions in Borderplex Community Health Workers: Impact of Age and Experience

**DOI:** 10.1177/21501319251394872

**Published:** 2025-11-23

**Authors:** Carla W. Irigoyen-Amparan, Gloria Alyssa Chavez-Saenz, Aditi Kaushal, Karen D. Gonzalez, Bibiana Mancera, Arunkumar Pennathur, Priyadarshini R. Pennathur

**Affiliations:** 1 University of Texas at El Paso, TX, USA

**Keywords:** burnout, community health workers, Borderplex, prevalence

## Abstract

**Introduction:**

The aim of this study is to examine the prevalence, extent, and characteristics of burnout among Community Health Workers (CHWs) in the US–Mexico Borderplex.

**Methods:**

CHWs in the Borderplex were recruited for a survey study assessing burnout using Maslach Burnout Inventory. Purposive and convenience sampling approaches were used to recruit participants. Descriptive and statistical analyses were conducted to assess prevalence and distribution of burnout by age and years of experience and burnout profiles of CHWs were generated.

**Results:**

The burnout prevalence among Borderplex CHWs is 42%. We found that CHWs in the Borderplex experience lower levels of burnout than other healthcare workers in the US based on comparison with normative values in the MBI, with older, more experienced CHWs reporting the lowest burnout. CHWs report lower mean EE (mean 15.34, SD 11.7) and DP (mean 3.16, SD 3.9) scores and moderate PA (mean 36.6, SD 9.5) scores. Age and Years of Experience impact CHW burnout with those in the 25 to 34 age group and with 0 to 5 years of experience reporting moderate to high burnout dimension scores.

**Conclusions:**

Our study advances our understanding of burnout among the previously unexplored population of Borderplex CHWs. Our findings suggest that a sense of personal accomplishment may significantly mitigate burnout. More holistic intervention approaches, considering work–life integration factors as well as personal and work-related factors, can improve well-being and prevent burnout among all healthcare workers.

## Introduction

Burnout significantly impacts healthcare workers, with recent studies indicating that over 50% report experiencing symptoms.^1-3^ Similarly, a 2019 report found that 35% to 45% of nurses and 40% to 54% of physicians in the United States experienced burnout over the past decade.^4^ Typically characterized by three dimensions—emotional exhaustion, depersonalization or cynicism, and a low sense of professional accomplishment^5^—burnout can affect job satisfaction, productivity,^6^ staff turnover, and the quality of patient care.^7^ As conceptualized by the Maslach Burnout Inventory (MBI), emotional exhaustion dimension indicates “feelings of being emotionally overextended and exhausted by one’s work”; depersonalization denotes “an unfeeling and impersonal response toward recipients of one’s service or care”; personal accomplishment indicates “feelings of competence and successful achievement in one’s work.”^8^ Burnout’s adverse consequences can include depression, anxiety, and suicidal ideation, especially among high-stress professionals such as surgeons.^9^ Costing an estimated $4.6 billion annually in the US due to reduced clinical hours and the need to replace staff,^1,10^ the significant impact of burnout on healthcare workers and the wider system underscores the need for interventions.

While burnout has been investigated among frontline healthcare workers, it remains relatively unexplored among other professionals, such as public health workers. Scales et al’s^11^ recent analysis revealed prevalent burnout among this group, identifying sleep quality and hours worked as significant predictors, and Stone et al^12^ found that 66.2% of public health workers experienced burnout during the COVID-19 pandemic. A systematic review by Spencer-Hwang et al^13^ highlighted overwork and lack of support as major risk factors for burnout among public health workers. These studies all indicate the potential for burnout among public health workers while highlighting the need for further research.

Understanding of burnout characteristics among community health workers (CHWs), a type of public healthcare worker, remains even more limited. CHWs play a key role in the healthcare system, providing culturally and linguistically appropriate health education and interventions, promoting health, coordinating care and case management, offering services such as health screenings, and providing social support, navigating complex systems to ensure that community members receive the services they need.^14^ During the pandemic, CHWs were also involved in community surveillance, contact tracing, risk communication, and home-based care,^15-17^ as well as promoting mask use, hand hygiene, social distancing, and testing and vaccination.^18^ The critical and challenging nature of CHWs’ work and its importance for enhancing community health and sustaining community-based interventions underscore the need to support their well-being and mitigate stressors to improve their effectiveness in delivering public health initiatives.^19^

CHWs face significant stressors, including long working hours and a workload similar to that of frontline healthcare workers.^19-21^ However, they also face unique challenges such as high anxiety levels and low job satisfaction, potentially impacting their productivity and self-esteem.^21^ Unlike frontline healthcare workers, CHWs typically operate in resource-limited communities with inadequate support systems and receive insufficient training and little recognition for their work.^22-25^ Many are uncompensated, serving only in a voluntary capacity. Furthermore, CHWs live within the communities they serve, blurring boundaries, increasing expectations, and intensifying stress.^20,26^ Frequently providing services in a humanitarian context or within underserved and vulnerable populations,^19,24^ CHWs are required to be highly adaptable to address complex cultural and health challenges, leading to significant emotional labor.^19,24^ Their emotional proximity to the community, coupled with the responsibility of linking them with health services, further adds to CHWs’ psychological burden.^20,24^

CHWs in any border region, or Borderplexes, face distinct challenges that can impact their well-being. While specific studies on Borderplex CHW burnout are lacking, research from low- and middle-income countries, which face similar socio-cultural, economic, and geopolitical challenges to Borderplexes, show that CHWs in these communities often face a high workload due to patient volumes and staffing shortages,^27,28^ yet receive little recognition for their work, increasing their risk of burnout.^26^ CHWs in the U.S.-Mexico border must also navigate cultural barriers and stigma associated with health challenges, creating the additional burden of promoting health within these communities while maintaining their trust.^29^ Additionally, systemic obstacles, such as language barriers, limited services, low health literacy, and a lack of culturally sensitive care, may arise, exacerbating CHWs’ feelings of inefficacy.^29,30^ Their exposure to socio-economic problems in their communities, can lead to emotional exhaustion and feelings of helplessness.^20,31^ Further, economic constraints and immigration barriers may limit CHWs’ paid employment opportunities, leading to job insecurity.^21,24,28,32^ The unique stressors CHWs experience potentially impact their well-being and the quality of care they provide,^33^ and make it crucial to characterize and address burnout, particularly among those in Borderplexes that have distinct socio-economic, cultural, and geopolitical influences.^33^

Despite the crucial role of CHWs and the unique stressors they face, the characteristics of burnout among this population and its prevalence are poorly understood. Ndulue et al’s^34^ systematic review captured some of the few studies that assessed the prevalence and characteristics of mental health issues among CHWs in low- and middle-income countries which indicated that CHWs experience mental distress, poor sleep, and an increased risk of burnout. In Brazil, 29.3% of CHWs exhibited symptoms associated with burnout,^35^ whereas other types of CHWs such as human service workers in Portugal and community social healthcare professionals in Spain reported low to moderate burnout.^36,37^ Focusing specifically on well-being during the pandemic, Yella and Dmello^38^ reported a 10.5% prevalence of burnout among CHWs in Andhra Pradesh, India. Rahman et al^22^ assessed predictors of burnout, suggesting that CHWs with higher anxiety and lower job satisfaction are more likely to experience burnout. While these studies somewhat elucidate the prevalence of burnout among CHWs, most focus on low- and middle-income countries, with only 1 study conducted in the US. Additionally, many recent studies have focused only on burnout during the pandemic,^38^ and few report prevalence rates among CHWs, an essential factor for characterizing burnout and developing interventions.

Notably, no studies have yet explored burnout among Borderplex CHWs—whether and to what extent these workers experience burnout remains unclear. To address this gap, we present findings from a survey study using the Maslach Burnout Inventory (MBI)^8^ that examined the prevalence, extent, and characteristics of burnout among CHWs in the US–Mexico Borderplex.

The aims of our study are to assess the prevalence, extent, and characteristics of burnout among CHWs in the US–Mexico Borderplex using the MBI, and to understand the impact of age and experience on burnout dimensions among CHWs.

## Methods

### Setting and Participants

This study was conducted in a University located in the Southwest, but the sample of CHWs were from El Paso County, TX and southern Doña Ana County, NM. The study was conducted between late Fall 2023 and Spring 2024. We used a combination of purposive and convenience sampling to recruit CHWs from local community organizations in the Borderplex. Our sample size was determined pragmatically rather than through an a priori statistical power calculation. The inclusion criteria required participants to be either currently serving or having previously served as CHWs, with no restrictions regarding years of experience (YOE) or compensation type.

The Institutional Review Board (IRB) approved this study in December 2023. Study details were disseminated by email to potential CHW participants through the program coordinator affiliated with the University and a local community health worker organization in the Borderplex region. We conducted the study in 2 phases. The first phase was part of a larger study which included follow-up interviews with CHWs held in-person or virtually to complete a survey assessing burnout. The program coordinator shared study information with all CHWs affiliated with the organization and mentioned that they should contact the study PIs directly if they were interested in participating. The second phase consisted of emails sent through the program coordinator to participants which included a link to the survey for them to complete. CHW participants completed the survey directly. We used a survey feature to ensure the same participant did not complete the survey twice. Our recruitment strategy, which combined purposive and convenience sampling across 2 study phases (in-person/virtual recruitment through a program coordinator, and direct email outreach), was designed to maximize participation while ensuring diverse representation. This paper reports only the survey findings from the 2 study phases and not interview findings.

### Tools

The survey captured demographic information (age, sex, ethnicity, and YOE) and included the Maslach Burnout Inventory (MBI), a validated survey instrument to assess an individual’s risk of burnout.^8^ The MBI assesses 3 dimensions of burnout: a 9-item emotional exhaustion (EE) scale, reflecting emotional draining after prolonged exposure to stress, with higher scores reflecting greater experienced burnout; a 5-item depersonalization or cynicism (DP) scale, indicating feelings of detachment from one’s surroundings, with higher scores reflecting greater experienced burnout; and a 8-item personal accomplishment (PA) scale, reflecting feelings of success in one’s work role, with lower scores reflecting greater experienced burnout. It is comprised of 22 statements about specific experiences rated on a 6-point Likert scale, with “0” indicating “never,” and “6” indicating “every day.” The survey’s cover page provided information regarding consent and study details.

### Experimental Procedure

In the first phase, the researcher opened the survey link for participants to complete before the interview. In the second phase, participants completed the survey independently using the link sent via email and did not participate in any follow-up interviews.

To avoid influencing participants’ responses, the study’s purpose was not disclosed to the participants, in accordance with MBI recommendations. We informed participants that the study was assessing job attitudes. The survey was available in both Spanish and English. Following completion of the survey, participants were debriefed on its true purpose and asked if they still consented to allowing their survey responses to be used as part of the study in light of the new information.

### Analysis

We analyzed participant demographics— gender, age, ethnicity, and YOE— using descriptive statistics to determine the means and distributions. CHWs were categorized into the following age groups: 18 to 24, 25 to 34, 35 to 44, 45 to 54, 55 to 64 and >65. Survey scores from the Spanish version were entered by the native Spanish-speaking members of the research team.

#### Burnout Prevalence and Comparison

To analyze burnout prevalence, we compared each participant’s total score for each dimension with the cut-off values provided in the MBI Manual,^8^ where a high score was defined as ≥27 for EE, ≥10 for DP, and between 0 and 33 for PA. CHW burnout prevalence was then calculated as the percentage of participants with a “high” score in at least 1 burnout dimension. This method facilitates differentiation between individuals with and without burnout. Additionally, we grouped participants with at least 1 “high” burnout dimension by age and YOE.

MBI has established normative values of the burnout dimensions for healthcare workers (EE = 22.19, DP = 7.12, PA = 36.53, derived from the MBI 4^th^ Edition Manual^8^). We compared our CHW sample means to these normative values using a t-test.

For each participant, each burnout dimension was categorized as low, moderate, or high based on the cut-off scores. Burnout profiles were generated based on combinations of low, moderate, or high categories for EE, DP, and PA. MBI provides 5 profiles based on these combinations: Engaged, Ineffective, Overextended, Disengaged, and Burnout. Our CHW sample did not always fit into these 5 profiles, necessitating the generation of 2 additional profiles: Mixed, where PA was always high, but EE and DP varied between low and moderate; and Drained and Detached, where EE and DP remained high and moderate, but PA varied between low and high. We also determined the distribution of burnout profiles by age and YOE.

#### Impact of Age and Years of Experience

We determined the mean of each burnout dimension for each age group and produced boxplots to visualize the distribution of these dimensions across age categories. To assess the impact of age on burnout dimensions, we performed an ANOVA at a 95% confidence interval.

We determined the mean for each burnout dimension, grouped by YOE, and performed a linear regression on these variables.

## Results

We aimed to assess the prevalence of burnout among CHWs in the Borderplex and generate burnout profiles for this population. Given the significant burnout among other healthcare workers, we hypothesized that CHWs, frequently working voluntarily with no or little compensation, would experience even greater burnout.

We analyzed descriptive statistics to describe the sample’s characteristics (Table 1). The study’s overall response rate was 43% (43/100), with 43 CHWs participating (females, male, and non-binary). Most (n = 42) identified as Hispanic or Latino. The mean YOE was 4.8 years, SD 6.8. The largest age groups were 55 to 64 (28%) and 25 to 34 (26%). Overall, EE (mean 15.34, SD 11.7) and DP (mean 3.16, SD 3.9) scores were considered low, and PA was moderate (mean 36.6, SD 9.5).

**Table 1.** Overall Demographics and Descriptive Statistics.

CHW demographics	Percentage
Age	
25-34	25.58
35-44	16.28
45-54	18.60
55-64	27.91
Other	11.63
Ethnicity	
Other	2.33
Hispanic or Latino	97.67
Years of experience	
0-5	72.09
5-10	16.27
Other	11.6
Burnout dimensions	Mean total score, SD
Emotional exhaustion	15.34, 11.7
Depersonalization	3.16, 3.9
Personal accomplishment	36.6, 9.5

### Burnout Prevalence Among CHWs in the Borderplex is 42% and is Lower Than Among Other Healthcare Workers Nationally

#### Burnout Prevalence

To assess the prevalence of burnout among CHWs, we analyzed the number of participants with a high score in at least 1 burnout dimension. We found that 42% (n = 18) of CHWs in our sample exhibited a high level of burnout in at least 1 dimension.

#### Burnout Prevalence Distribution by Age and Years of Experience

To understand how burnout prevalence varies by age and YOE, we categorized CHWs reporting at least 1 high-scoring burnout dimension (n = 18) by these variables. We found that CHWs in the 25 to 34 age group (Table 2) and those with 0 to 5 YOE (Table 3) more frequently reported high burnout in at least 1 dimension. Of the 18 CHWs, 13 reported low PA, 4 reported high DP, and 7 reported high EE. Those reporting low PA have between 0 and 10 YOE, indicating that younger and less experienced CHWs are more likely to report high burnout, with low PA being the most influential factor.

**Table 2.** CHWs With at Least 1 High Burnout Dimension, Categorized by Age.

Age categories	Distribution of CHWs with at least 1 dimension of burnout high	Count of CHWs with low PA	Count of CHWs with high EE	Count of CHWs with high DP
18-24	2	2	0	0
25-34	8	6	3	3
35-44	3	2	3	0
45-54	2	1	1	0
55-64	2	2	0	0
>65	1	0	0	1
**Total**	18	13	7	4

**Table 3.** CHWs With at Least 1 High Burnout Dimension, Categorized by YOE.

Years of experience	Distribution of CHWs with at least 1 dimension of burnout high	Count of CHWs with low PA	Count of CHWs with high EE	Count of CHWs with high DP
0-5	15	11	5	2
5-10	2	2	1	1
15-20	1	0	0	1
Total	18	13	6	4

#### Comparison Between Sample and Normative Means

The comparison between our sample means and the normative values provided by MBI revealed significant differences for EE (*P* *=* .*0004*) and DP (*P* *=* .*00*). This indicates that the burnout dimensions reported by CHWs in our sample are significantly lower than the normative values for healthcare workers.

#### Burnout Profiles

**Figure 1.**
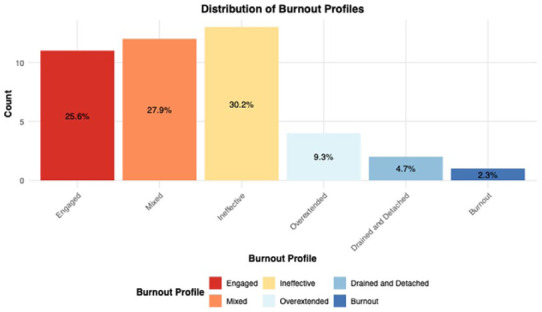
Distribution of burnout profiles among CHWs.

The majority of CHWs fall into the Ineffective (30%), Mixed (28%), and Engaged (26%) profiles, and very few exhibit burnout (2%; see Figure 1). The Mixed profile indicates that CHWs experience some EE and DP while maintaining effectiveness and engagement. Similarly, participants with the “Drained and Detached” profile demonstrate high EE, moderate DP, and high PA. These findings indicate low burnout in general, but with some CHWs reporting low PA.

### Older CHWs Report Lower Burnout

#### Burnout Profile Descriptions by Age

**Figure 2.**
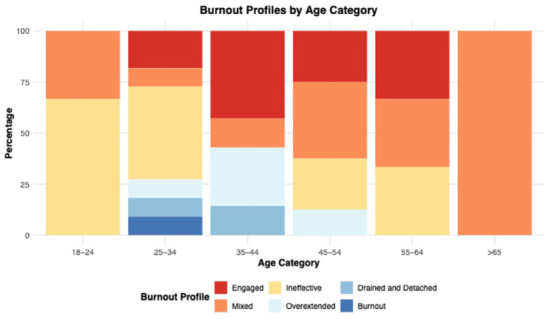
Distribution of burnout profiles among CHWs by age.

We computed the distribution of burnout profiles to identify the percentage of participants in each profile categorized by age. The Engaged profile is more prevalent in the 55 to 64 age group, whereas Ineffective and Burnout are more common in the 25 to 34 age group (Figure 2). Additionally, 28% of all participants exhibit Mixed profiles, which was more common among middle-aged and older groups. These findings suggest that age may influence burnout.

#### Descriptive Statistics of Burnout Dimensions by Age

To identify which age groups experience the least burnout, we calculated the means of burnout dimensions across age categories. We found that CHWs aged 55 to 64 years (Table 4) show the lowest EE (mean 8.5, SD 7.2) and DP (mean 8.5, SD 3.48) and the highest PA (mean 40.25, SD 7.6), indicating low burnout among this age group.

**Table 4.** Means and SD of Emotional Exhaustion, Depersonalization, and Personal Accomplishment Scores by Age.

Age categories	Count of CHWs	YOE, mean (SD)	EE, mean (SD)	DP, mean (SD)	PA, mean (SD)
18-24	3	1.25 (1.52)	18.67 (11.85)	4.67 (2.08)	28.33 (10.12)
25-34	11	1.81 (1.61)	19.18 (13.83)	4.73 (5.3)	30.459 (10.59)
35-44	7	2.70 (2.01)	22.43 (12.31)	1.71 (2.21)	39.29 (8.85)
45-54	8	7.5 (9.02)	11 (9.9)	2.38 (3.11)	39.63 (8.03)
55-64	12	5.61 (8.3)	8.5 (7.2)	2.42 (3.48)	40.25 (7.6)
>65	2	17.5 (3.54)	23 (4.24)	5 (7.07)	40.5 (0.71)

#### Distribution of Burnout Dimensions by Age

**Figure 3.**
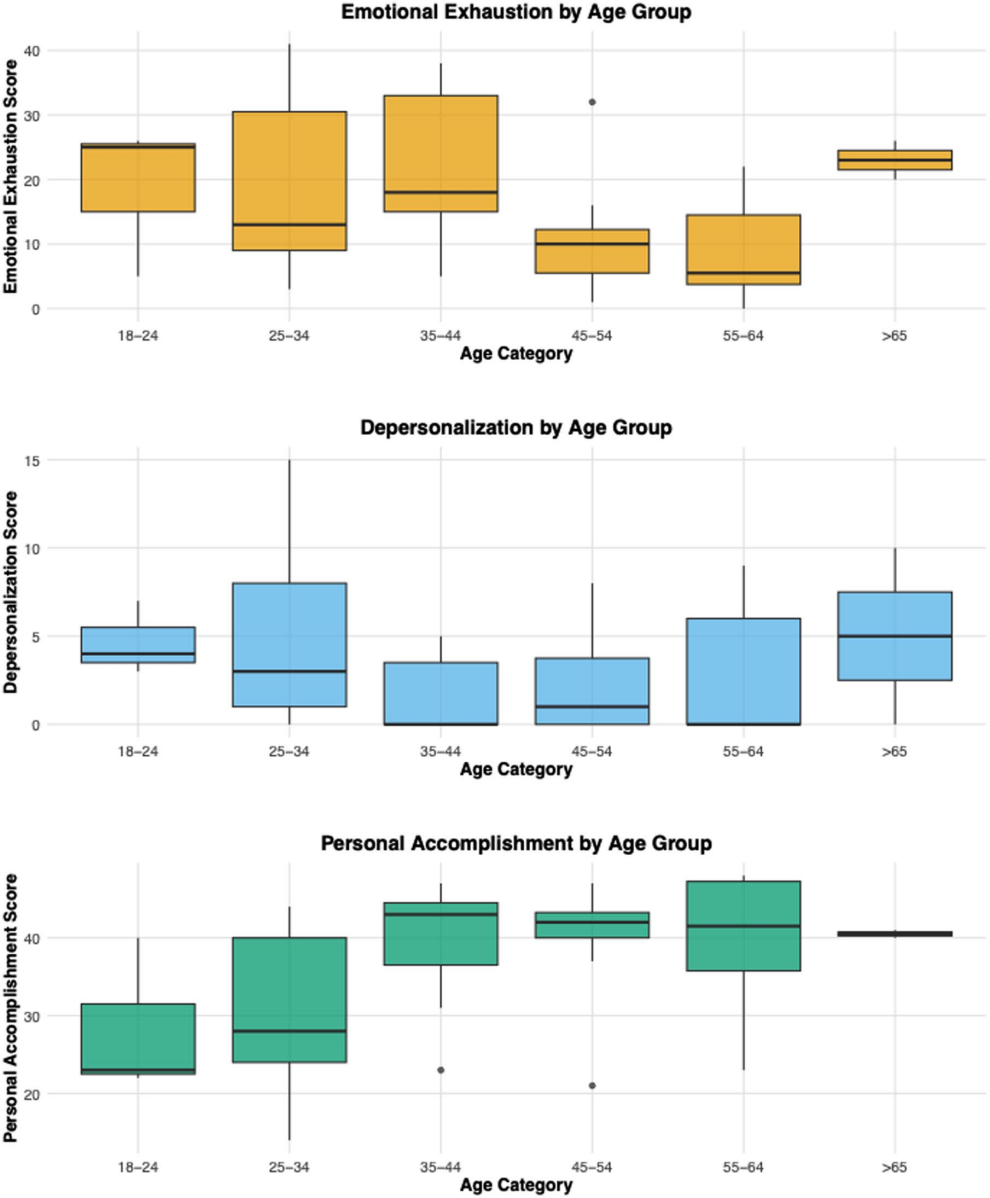
Boxplots of emotional exhaustion, depersonalization, and personal accomplishment scores for CHWs categorized by age.

Figure 3 presents boxplots illustrating differences in the distribution of each burnout dimension by age group. The 18 to 24 age group had a median EE of 25 and a median DP of 4, whereas the 55 to 64 age group had a median EE of 5.5 and a median DP of 0. The 18-24 (median = 23) and 25 to 34 age groups (median = 28) had the lowest PA scores. The >65 age group had median EE and DP scores of 23 and 5, respectively, but a median PA of 40.5. These results indicate that EE and DP tend to decrease with age, whereas PA increases. While the >65 age group reported high EE and DP, their high median PA may be indicative of a protective effect.

#### ANOVA of Age and Burnout Dimensions

The ANOVA assessing the impact of age on burnout dimensions revealed that the effects on EE and PA were not statistically significant (*F* = 2.33, *P* *=* .*062* and *F* = 2.4, *P* = .056, respectively*)* but approached significance, indicating a marginal impact of age on EE and PA (Table 5).

**Table 5.** ANOVA of emotional exhaustion, depersonalization, and personal accomplishment scores and age.

Burnout dimension	*F* value	*P*-value
Emotional exhaustion	2.32866528	.0618221
Depersonalization	0.8685399	.51142873
Personal accomplishment	2.39947716	.0555684

### More Experienced CHWs Report Lower Burnout

#### Burnout Profile Descriptions by Experience

**Figure 4.**
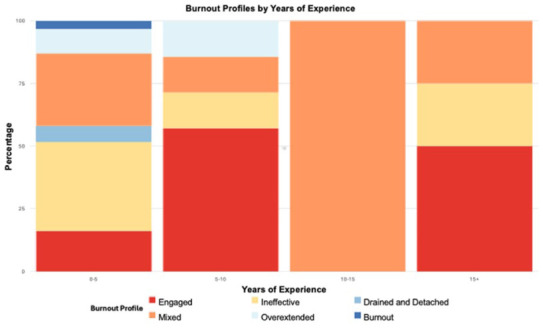
Distribution of burnout profiles among CHWs by years of experience.

We computed the distribution of burnout profiles to identify the percentage of participants in each profile categorized by years of experience (Figure 4). The Engaged (57%) and Overextended (14%) profile is more prevalent in CHWs with up to 10 years of experience, whereas Ineffective (35%), Drained and Detached (6%), Mixed (29%) and Burnout (3%) profiles are more prevalent in those with less than 5 years of experience. These findings suggest that experience may influence burnout.

#### Descriptive Statistics of Burnout Dimensions by Years of Experience

We computed the mean scores of burnout dimensions to assess their relationship with YOE (Table 6). Our results reveal a mean EE of 16.1 for CHWs with 0 to 5 YOE, and a mean EE of 9.5 for those with 15 + YOE. The mean PA was 35.13 for the 0 to 5 YOE group and 41 for the 15 + YOE group. The mean DP hovers around 3 for all groups. These findings indicate that mean EE scores decrease, and mean PA scores increase with increasing YOE, while DP scores remain low across all levels of experience.

**Table 6.** Means and SD of Emotional Exhaustion, Depersonalization, and Personal Accomplishment Scores by Years of Experience.

Years of experience	n	EE, mean (SD)	DP, mean (SD)	PA, mean (SD)
0-5 years	31	16.1 (12.3)	3.23 (3.65)	35.13 (10.29)
5-10 years	7	13.86 (11.11)	3.71 (4.96)	40.29 (7.61)
10-15 years	1	26 (NA)	0 (NA)	41 (NA)
15 + years	4	9.5 (7.94)	2.5 (5)	41 (3.16)

#### Linear Regression of Burnout Dimensions and Years of Experience

**Figure 5.**
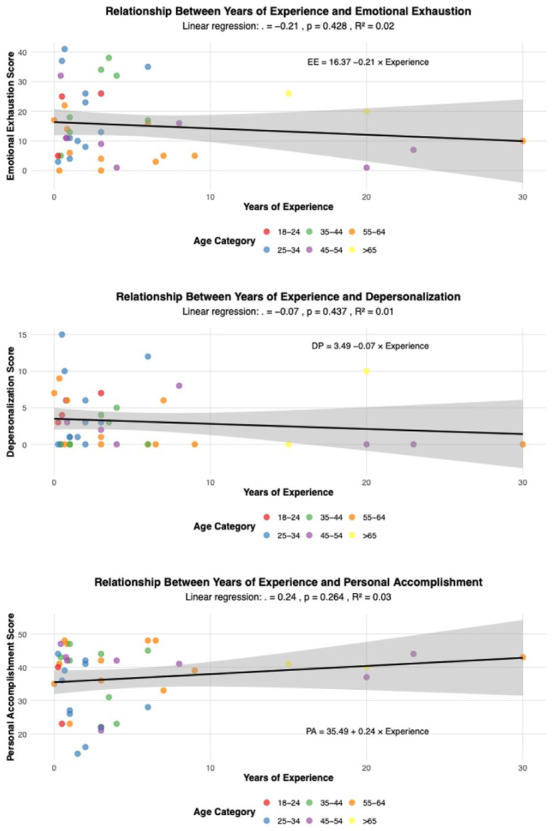
Linear regression between years of experience and emotional exhaustion, depersonalization, and personal accomplishment scores.

The linear regression assessing whether YOE predicts burnout scores revealed that YOE has a negative relationship with EE (*P* *=* .*428*, *R*² = 0.015) and DP (*P* *=* .*437*, *R*² = 0.015) but a positive relationship with PA (*P* *=* .*264*, *R*² = 0.03; Figure 5). None of these findings were statistically significant. The PA intercept suggests that new CHWs start with moderate levels of personal accomplishment.

## Discussion

This study assessed the prevalence and characteristics of burnout among US–Mexico Borderplex CHWs, using the MBI to assess burnout. This research addresses a significant gap in the literature by providing prevalence rates on burnout in this population. We found that US–Mexico Borderplex CHWs experience lower levels of burnout than other healthcare workers in the normative sample provided by MBI, with older, more experienced CHWs reporting the lowest burnout.

The 42% burnout prevalence among Borderplex CHWs found in this study is lower than the 50% or more recently reported among frontline healthcare workers in general.^1-3^ This is supported by the lower mean EE and DP scores, and moderate PA scores of our CHW sample compared to the MBI’s normative values for healthcare workers. We propose that this difference stems from the distinct challenges faced by the different types of healthcare workers. Unlike frontline healthcare workers, who are encumbered with the financial burden of educational loan repayments, heavier workloads, high-pressure environments, constraints from federal requirements, and potential litigation, CHWs can operate with greater autonomy and carry no professional liability. This difference in how they operate can significantly lower emotional exhaustion. Unbound by constraints or policies, they offer services to their communities at no cost, operate with a “volunteer” ethos, and perceive compensation only as a bonus—they instead find meaning in the legitimacy of their titles and certifications, regarding them as recognition of their value to the community. These factors can reduce cynicism about one’s work and increase feelings of personal accomplishment based on their perceived value to the community. We posit that these distinct characteristics and demands contribute to an overall lower emotional exhaustion, depersonalization and moderate personal accomplishment, and a lower prevalence of burnout.

Burnout prevalence among Borderplex CHWs appears to be moderate compared to that among CHWs in other countries. However, direct comparisons are challenging due to the limited number of studies reporting prevalence rates and variations in instruments and sample characteristics. Compared to previous studies reporting prevalence rates ranging from 10% to 30% or more,^20,22,34-39^ our finding of 42% seems to be slightly higher. This marginally higher prevalence may be due to the unique stressors of the US–Mexico Borderplex, but more research is needed to understand the contributory factors, and more consistent reporting of prevalence rates is required to facilitate comparison.

Our findings show that age impacts EE, DP, and PA burnout dimension scores among Borderplex CHWs, as well as a higher burnout prevalence in younger CHWs. Those in the 25 to 34 age group report moderate to high burnout dimension scores, indicating that CHWs in this age group are at higher risk of burnout. Middle-aged and older CHWs exhibit comparatively less EE and DP, whereas younger CHWs exhibit higher EE. Previous research has demonstrated a relationship between age and burnout.^40-42^ Higher burnout in younger CHWs may stem from their relative lack of impactful experiences, which could limit their self-efficacy and the perceived value of their work. Additionally, they may lack the emotional maturity and emotional intelligence to handle complex community challenges compared to their older counterparts, who may have developed these skills through their community and life experiences. Emotional intelligence inversely relates to burnout, which could explain the higher EE among younger CHWs.^43-46^

Older workers may experience different levels of job satisfaction and anxiety from their younger colleagues,^22^ in turn potentially affecting their well-being. Older CHWs may have also developed coping skills through accumulated life and work experiences to provide a buffer against burnout,^47^ helping them modulate their EE and DP.^48^ While younger CHWs gain emotional intelligence, coping skills and task-specific knowledge by shadowing experienced CHWs in the field, incorporating emotional intelligence training for new CHWs during onboarding could help accelerate this learning process and provide evidence-based coping strategies for all CHWs.

Our findings also demonstrate a positive impact of YOE on burnout, with higher prevalence among less experienced CHWs. Those with 0 to 5 YOE report moderate to high burnout dimension scores, indicating that the less experienced CHWs are at a greater risk of burnout. YOE has a negative relationship with EE and DP, further illustrating that increase in YOE is predictive of decrease in burnout experienced. Some studies suggest that burnout increases between 5 and 10 YOE and then decreases after 10 years,^43,44^ supporting our findings. Less experienced CHWs may have lower socio-economic status, with additional financial stress and job insecurity leading to a higher risk of burnout.^24^ More work experience can help CHWs develop better coping skills, which potentially explain why the more experienced CHWs in our study had lower burnout scores. Conversely, other studies have found no significant relationship between experience and burnout,^35^ instead suggesting that more experienced CHWs report lower satisfaction and higher burnout due to unmet needs.^22,24^ Organizations should tailor CHWs’ work activities based on their experience, complementing them with organizational support to reduce burnout in the long term. Additionally, longitudinal studies are needed to understand the association between burnout, YOE, and job contexts.

Although middle-aged and older CHWs had lower EE and DP, the oldest CHWs in our sample (>65 years) reported high EE and high PA. While age-related fatigue could explain high EE in this age group,^40,41^ older workers and those with many YOE may have experienced prolonged exposure to stressors such as long hours, few resources, and emotional labor, adversely impacting their long-term well-being. For example, Telles and Pimenta^24^ reported that CHWs with more than 6 YOE exhibited higher EE and DP. Research suggests that individuals performing a significant amount of care work and emotional labor may experience compassion fatigue if not adequately managed.^49^ These findings indicate that long-term exposure to stress as well as age- and experience-related factors may increase the risk of burnout.

Despite the lower prevalence of burnout among Borderplex CHWs, many exhibit higher EE scores. A CHW’s daily activities involve substantial emotional labor, and research indicates that deep emotional labor is associated with lower job satisfaction and higher burnout among them.^48^ Other work-related factors, including higher anxiety levels and lower job satisfaction, also increase the risk of greater EE.^22,50^ Furthermore, the professional skills needed to navigate stigma around health problems may introduce significant stress^37,51^ especially long-term. CHWs may also not know how to relax and unplug from work and life, which could adversely impact their well-being.

EE among Borderplex CHWs may be related to cultural factors that can exacerbate the need to perform emotional labor. CHWs often serve as “cultural brokers” in their communities,^52^ engaging in cross-cultural interactions to build trust and rapport.^53,54^ The social and cultural norms of CHWs’ communities also shape their coping skills and work environment.^55^ Working within their communities often leads to a blurring of personal and professional boundaries,^26^ and CHWs may experience feelings of inefficacy if they cannot resolve community issues.^26,35^ The need for CHWs to function within the cultural context of a community can introduce stressors that engender feelings of low PA. This underscores the need to consider the cultural context and emphasize strategies to enhance feelings of personal accomplishment.

PA can serve as key protective factor against burnout, and the majority of CHWs in our sample exhibited moderate to high levels of it. Our findings also show that CHWs have a moderate PA score compared to other healthcare workers, and their PA increases with YOE. Additionally, newer CHWs begin with moderate PA. For many CHWs in our sample, we anecdotally know that certification and working for the university instill a sense of pride and accomplishment, evidenced by their moderate to high PA scores. Personal accomplishment enhances self-efficacy and satisfaction, reducing the risk of burnout.^24,56^ While surface-level emotional labor results in higher job satisfaction and higher PA, deep emotional labor can have a negative impact.^48^ Emotional intelligence training for CHWs can help reduce the effects of emotional labor, and supportive work environments that enable skill development and offer feedback can boost PA.^57^ Cultivating authentic ways to recognize CHWs and increase their PA and self-efficacy can help boost the moderate PA and may protect against the EE inherent in their roles. Organizational initiatives should amplify recognition and value, clarify the connections between CHWs’ work and community health outcomes to enhance compassion satisfaction, and provide opportunities for professional growth. Celebratory events and genuine acknowledgment of their contributions can enhance their self-efficacy, social well-being,^58^ and value congruence,^59^ and help connect their work to the community’s perceived value. Improved integration of CHWs within formal healthcare organizations can further enhance CHWs’ effectiveness and increase their sense of value.^60^

The concepts of resilience and compassion satisfaction can also help explain why CHWs report lower burnout rates. CHWs often experience personal challenges or have overcome adversity, which may have helped them be resilient. Research demonstrates that psychological resilience mitigates burnout.^36,47,61^ Given this, organizations can develop a culture of support to encourage resilience-building^19,62^ to help alleviate the burden of emotional labor.

Healthcare workers generally exhibit a high level of compassion satisfaction as they gain fulfillment and satisfaction from helping others.^39,63^ Experience, perceived social support, and work environments that align with one’s personal values and goals can lead to higher compassion satisfaction.^64,65^

High levels of compassion satisfaction are associated with resilience and contentment, which, in turn, relate to discovering one’s calling and living authentically.^66^ CHWs possess extensive lived experiences that enable them to relate authentically to community issues and therefore become trusted in their communities.^60,67^ Driven by a desire to support their communities^68^ CHWs’ complement their intrinsic altruism with problem-focused coping strategies,^24^ which can act as a buffer against stress.^68^ The Spanish saying “Una promotora no se hace, nace,” meaning “A CHW is not made, but is born with the will or desire to help,” captures the essence of the characteristics needed for CHW work. These inherent traits can mitigate burnout by enhancing compassion satisfaction.^55,69^

However, scholars also call for interventions to address psychosocial issues among CHWs rather than solely relying on their altruistic behavior or inherent resilience.^35,70^ Research indicates that perceiving their work as a “paid job” rather than an altruistic endeavor could help CHWs approach challenges rationally and facilitate the adoption of surface-level emotional labor.^48^ Organizational initiatives that complement altruistic behavior and develop resilience, contentment, and compassion satisfaction can strengthen the positive effects of community work.

Our study has some limitations. While it included 43 CHW participants from the Borderplex region, a larger sample from similar Borderplex regions could yield more conclusive results. The unique characteristics of the Borderplex region—including its binational context, the diversity in CHW roles, and the challenges inherent in recruiting from border populations—meant that a pragmatic approach such as purposive and convenience sampling to sample size was necessary to ensure study feasibility while maintaining methodological rigor. The resulting sample enhances the local relevance and applicability of our findings to CHWs working in similar border contexts.

In aiming to understand the prevalence of burnout among Borderplex CHWs and explore how different burnout dimensions manifest, we only used the MBI to assess contributing factors. While the MBI is a validated instrument for assessing burnout, other instruments measuring slightly different dimensions of burnout exist. Using these additional instruments could provide insights on burnout dimensions not measured by MBI and allow for cross-validation of results.

Our findings offer several directions for future research. Future work could focus on elucidating the contributors to burnout among Borderplex CHWs, particularly among the younger professionals who exhibited a higher risk of burnout in the present study. Additionally, the incentives for undertaking this work may be inadequate given the job insecurity that younger professionals potentially face. Organizational and policy initiatives aimed at promoting CHWs’ professional development and funding^71^ to offer financial incentives may provide a sustainable income source for CHWs while preparing them for leadership roles in this field.

Researchers should also investigate the contributors to burnout and diminished well-being across geographical regions, especially border regions. Conducting geographically diverse studies to examine the unique challenges and work system elements specific to different cultural settings is warranted to explore whether a universal strategy to enhance well-being and decrease burnout is effective. Future work should develop interventions tailored to cultural and community contexts of CHWs to enhance and sustain their health and well-being.

Work system elements, including organizational policies, leadership support, workload, resources for community-based work, training, and recognition and reward systems, can impact EE, DP, and PA. Future research could use systems-based frameworks such as the Systems Engineering Initiative for Patient Safety^72^ and the job demands and resources model^73^ to assess work system contributors to burnout among Borderplex CHWs and develop strategies to address them. Additionally, longitudinal studies could improve understanding both the contributors to burnout and their evolution over time, as well as how various organizational and personal coping strategies may mitigate their effects. Finally, studies could develop predictive models, identifying clear markers along the trajectory to burnout, to facilitate prevention efforts.

In summary, our study advances our understanding of burnout among the previously unexplored population of Borderplex CHWs in their unique cultural, geopolitical, and socio-economic setting. Our findings suggest that a sense of personal accomplishment may significantly mitigate burnout. However, realizing this as a mitigation strategy will require the implementation of targeted organizational interventions. We recommend that more holistic intervention approaches, that take into consideration work–life integration factors as well as personal and work-related factors, could prove more effective in improving well-being and preventing burnout among all healthcare workers.
